# Comprehensive Clinical, Serological, and Molecular Biomarker Profiling of Primary Sjögren’s Syndrome: A Single-Center Cohort Study in Northeastern Romania

**DOI:** 10.3390/ijms26136327

**Published:** 2025-06-30

**Authors:** Alexandru Lodba, Codrina Ancuta, Diana Tatarciuc, Magda Ecaterina Antohe, Ana Maria Fatu, Luciana-Oana Lodba, Cristina Iordache

**Affiliations:** 1 Faculty of Dental Medicine, Grigore T. Popa University of Medicine and Pharmacy, 700115 Iasi, Romania; lodba.alexandru@d.umfiasi.ro (A.L.); diana.tatarciuc@umfiasi.ro (D.T.); magda.antohe@umfiasi.ro (M.E.A.); ana.fatu@umfiasi.ro (A.M.F.); marina.iordache@umfiasi.ro (C.I.); 2 Department of Rheumatology, Faculty of Medicine, Grigore T. Popa University of Medicine and Pharmacy, 700115 Iasi, Romania; 3 Department of Medicine Specialties, Faculty of Medicine, Grigore T. Popa University of Medicine and Pharmacy, 700115 Iasi, Romania; 4 Department of Implantology, Removable Dentures, Technology, Grigore T. Popa University of Medicine and Pharmacy, 700115 Iasi, Romania; 5 LC Dental Orthoffice S.R.L., Zimbrului Street, bl. 2B, sc. A, ap. 3, 725300 Gura Humorului, Suceava County, Romania; lucianacondur@gmail.com; 6 Ergonomics Discipline, Faculty of Dental Medicine, Grigore T. Popa University of Medicine and Pharmacy, 700115 Iasi, Romania

**Keywords:** primary Sjögren’s syndrome, salivary flow, autoantibodies, biomarker-driven therapy

## Abstract

Primary Sjögren’s syndrome (pSS) exhibits considerable clinical and immunological heterogeneity, complicating personalized management. We aimed to delineate the demographic, functional, serological, histopathological, and therapeutic features of a Romanian pSS cohort and to identify biomarker–treatment correlations that could inform patient-oriented strategies. Thirty-two patients meeting the 2016 ACR/EULAR classification criteria for pSS were retrospectively analyzed. Data collected included demographics, autoantibody profiles (Anti-Ro/SSA, Anti-La/SSB, ANA, RF, Anti-CCP), immunoglobulin levels, complement consumption (C3/C4), minor salivary gland biopsy (focus score), salivary flow tests, and systemic inflammation markers (CRP). Pearson correlation matrices were constructed to explore the associations between serological markers and prescribed therapies. The cohort was predominantly female (87.5%) with a mean age of 52.8 ± 9.9 years. Seropositivity rates were 50% for Anti-Ro/SSA, 77% for Anti-La/SSB, and 40% for ANA. Clinically significant glandular dysfunction was evident in 65% of patients (unstimulated flow ≤ 0.1 mL/min), and all biopsies demonstrated focus scores > 1. Methotrexate use correlated strongly with Anti-Ro/SSA and Anti-La/SSB positivity (*p* ≤ 0.05), indicating its targeted application in seropositive sub-phenotypes. Conclusion: These findings underscore the immunologic and clinical diversity of pSS and support a biomarker-driven, multidisciplinary framework for personalized treatment. Larger prospective and multicenter studies are warranted to validate these correlations and to refine precision medicine approaches in pSS.

## 1. Introduction

Sjögren’s syndrome (SS) is a chronic autoimmune disease characterized by salivary and lacrimal gland dysfunction and atrophy [1,2]. In 1932, Danish ophthalmologist Henrik Sjögren described clinical and histological findings in 19 women, identifying a trio of keratoconjunctivitis sicca, xerostomia, and rheumatoid arthritis (RA). Sjögren subsequently introduced the name keratoconjunctivitis sicca to distinguish this condition from vitamin A deficiency (xerophthalmia) [3]. The disease is complex and intricate, often also affecting glands from various physiological systems, such as the gastrointestinal, respiratory, skin, and vaginal systems [1,4]. SS may present as a primary disorder (i.e., primary SS (pSS)) independent of other diseases or as secondary SS, associated with other systemic autoimmune rheumatic diseases, notably RA and systemic lupus erythematosus (SLE). Inflammatory T-cell infiltration of exocrine glands, especially the lacrimal and salivary glands, is a key characteristic of this condition. This leads to the characteristic sicca syndrome, which consists of dryness of the eyes and mouth. Furthermore, SS may impact many organs and systems, particularly in its primary manifestation, resulting in a diverse array of clinical features that can be categorized into exocrine glandular and extraglandular symptoms [5].

From an epidemiological point of view, pSS can be described by two important indicators. Incidence quantifies the chance of new pSS cases arising during a designated timeframe, whereas prevalence denotes the proportion of a population diagnosed with the condition. According to a worldwide epidemiological study, the incidence rate of SS is 6.92 per 100 000 person-years, and the prevalence rate is 60.82 cases per 100 000 inhabitants [1]. This disease affects women more than men, with a female/male ratio of 9:1. Typically it affects middle-aged women, between 40 and 60 years old. Although pSS is classified as a dental illness, it frequently affects other organs as well, resulting in vaginal dryness and arthralgia [1]. This gradually advancing autoimmune condition is marked by lymphocytic infiltration of the exocrine glands and considerable impairment of secretory function, resulting in xerostomia [6] and xerophthalmia [7].

**Figure 1:**
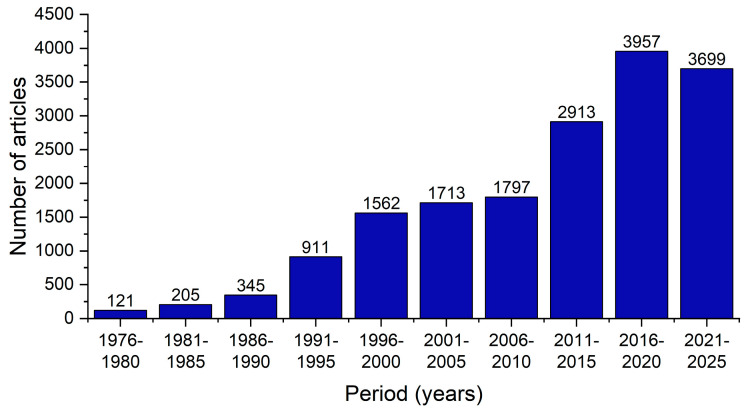
The number of articles published on Sjögren’s syndrome from 1976 to the present, sorted into five-year intervals, as determined by a Web of Science query (May 2025).

Primary SS proves to be a significant social and economic burden on our society, affecting the patients’ quality of life and impairing their daily activities and overall well-being [1]. The increasing scientific interest in this disease, particularly in the past decade, serves as evidence of its significance (Figure 1).

From a diagnosis point of view, SS has common symptoms with many other conditions, leading to a high chance of incorrect diagnosis. Thorough diagnosis involves assessing symptoms, the detection of specific antibodies, and salivary gland biopsy [8]. The currently used criteria are the American–European consensus published in 2002 [9]. Suspicions of SS should arise in individuals with chronic symptoms of xerophthalmia and/or xerostomia, parotid gland hypertrophy, or atypical outcomes in specific serological tests. These tests check for rheumatoid factor (RF), antinuclear antibodies (ANA), and Anti-Ro/SSA antibodies, either with or without Anti-La/SSB antibodies.

This study aims to comprehensively characterize the clinical and immunologic profiles of pSS patients and to define key correlations among histopathological findings, serological biomarkers, salivary dysfunction, and treatment responses, thereby elucidating underlying disease patterns.

## 2. Results

### 2.1. Patient Demographic Features

**Figure 2:**
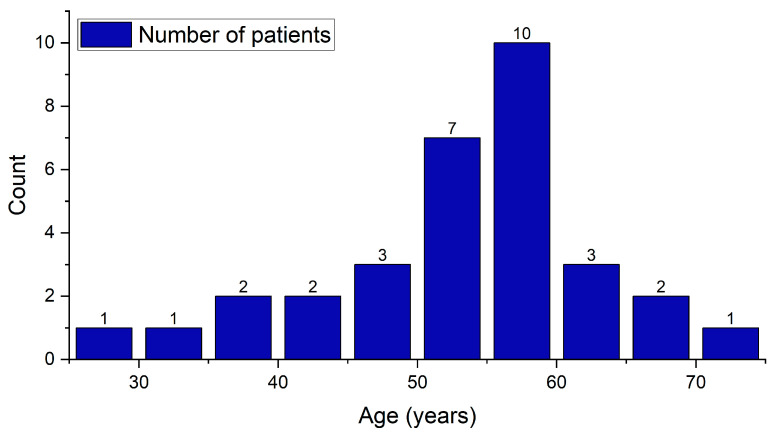
Patients’ age frequency distribution.

The study cohort is composed of 76 patients who were evaluated, and 32 of them were diagnosed with pSS. The bulk of these patients are female, comprising 28 (87.5%) patients. The ratio of females to males is 7:1. Regarding the age of the individuals, the youngest patient is 27 years old, while the oldest is 72. A considerable age gap exists between the average age of men, which is 61.5 ± 2.8 years, and the average age of women, which is 51.5 ± 9.8 years. The average age of all patients is measured at 52.8 ± 9.9 years (Table 1). The overall shape of the patients’ age distribution (Figure 2) appears to be right-skewed, meaning there are more patients in the middle to older age ranges. The majority of patients fall in the 50–60 age group, forming the high peak of the distribution chart, which aligns with the typical demographic patterns of Sjögren’s syndrome.

The normality tests conducted of patient’s age data were the Shapiro–Wilk (*p* = 0.34), Kolmogorov–Smirnov (*p* = 0.38), and Anderson–Darling (*p* = 0.15) tests. All tests report non-significant *p*-values (*p* > 0.05), suggesting that the null hypothesis of normality cannot be rejected. These results indicate that the age data are likely normally distributed, significantly drawn from a normally distributed population.

### 2.2. Serological Biomarkers

**Figure 3:**
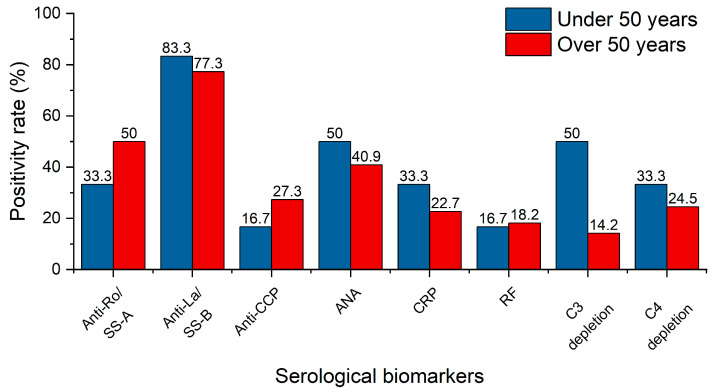
Serological biomarker positivity rates for the two study groups.

Of the 32 patients involved in this investigation, 28 underwent comprehensive serological biomarker analysis. The patients were categorized into two groups based on age: under and above 50 years, with the results provided separately (Figure 3). The most common autoantibodies in patients with SS are Anti-Ro/Sjögren’s syndrome A (SS-A) and Anti-La/Sjögren’s syndrome B (SS-B). The older group presented a higher positivity percentage for Anti-Ro/SS-A, suggesting a potential increase in autoimmune activity with age or with prolonged disease course. Anti-La/SS-B is a core biomarker of SS and is often used in its diagnose and classification. Anti-La/SS-B has a high positivity rate among patients, suggesting it is a significant disease marker. The autoantibody prevalence levels are high in both age groups, indicating it is a stable and early marker of the disease. It is also closely related to pSS. Anti-cyclic citrullinated peptide antibodies (Anti-CCP) and RF are strongly related to RA. Since their positivity rates are low, it suggests that most patients are likely to have pSS. ANA is a nonspecific autoimmune marker found in multiple autoimmune diseases (e.g., lupus, systemic sclerosis); therefore, it is less informative than Anti-Ro/SS-A or Anti-La/SS-B when analyzing pSS patients. Nevertheless, higher ANA positivity at younger ages suggests that pSS starts with a nonspecific immune dysregulation that evolves to the presence of more specific Anti-Ro and Anti-La antibodies. As a general inflammation indicator, C-reactive protein (CRP) is typically elevated in systemic inflammation without major significance in pSS. Its prevalence is relatively low, suggesting the localized character of the disease, rather than a systemic condition. Lower levels in older patients may be an indicator of inflammatory adaptation over long periods of time. Complement components 3 and 4 (C3 and C4) play a key role in the immune system, and the low levels of these complement system proteins suggest a chronic immune activation. Half of the below 50 years old patients had C3 depletion. This overreactive complement behavior suggests recent or ongoing immune complex formation and thus complement consumption. Older patients show lower C3 depletion, alluding to the idea that the immune system adapts its complement activity, leading to lower levels of both immune complex deposition and consumption. On the other hand, C4 depletion shows lower depletion rates for both age groups.

### 2.3. Salivary Tests

The patients underwent objective assessment of their salivary gland function, including whole stimulated and unstimulated salivary flow rate (mL/min), pH, and buffer capacity (mmol/L). These tests revealed serious modifications in saliva, both quantitatively and qualitatively. An unstimulated whole salivary flow rate (UWSFR) of lower than 0.1 mL/min was found in 20 patients, representing 62.5% of the study cohort. These patients meet the threshold defined by the 2016 ACR/EULAR classification criteria for pSS. Stimulated whole salivary flow rates (SWSFRs) have a broader range of values with a maximum of 2 mL/min. A reduced SWSFR (≤0.7 mL/min) was found in nine patients (28%). Patients show variable degrees of residual gland function, without a significant correlation between the two salivary flow rates. The values of the UWSFR are not reliable predictors of the values of the SWSFR, as some patients have abundant stimulated secretion despite having a noticeably reduced baseline flow rate. The other 12 patients who did not meet the UWSFR threshold defined by the 2016 ACR/EULAR classification criteria for pSS showed low to normal flow rates ranging from 0.2 to 0.7 mL/min, with an average of 0.4 mL/min. Additionally, all 12 patients presented normal stimulated whole salivary flow rates with values above the hyposalivation threshold of 0.7 mL/min, ranging from 0.9 to 2 mL/min, with an average value of 1.3 mL/min.

**Figure 4:**
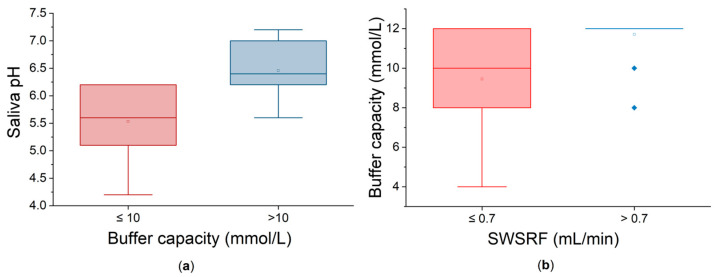
(**a**) saliva pH as a function of buffer capacity; (**b**) buffer capacity as a function of SWSFR.

Salivary pH values of ≤6 were found in 10 patients (31.25%). This acidic environment could lead to dental demineralization and mucosal irritation. Additionally, five patients (15.62%) exhibited decreased buffer capacity with values below 10 mmol/L, further compromising the protecting properties of the saliva. As expected, patients with low buffer capacities also had low pH values. In order to further analyze the interrelationship between pH and buffer capacity, the patients were divided into two groups based on whether their buffer capacity was low (≤10 mmol/L) or not. Statistically, at the 0.05 level, there is a highly significant difference between the two groups (Figure 4a). There are no major outliers visible. The buffer capacity factor used to divide the groups has a significant effect on the outcome, meaning that it is a clinically meaningful criterion. Causal and functional influences between these parameters can be observed.

On the other hand, dividing the patients based on their SWSFRs leads to two groups with statistically significant different values of buffer capacity (Figure 4b). Patients with normal/mild dysfunction (SWSFR > 0.7 mL/min) exhibit significantly higher and consistent salivary buffer capacity than the patients with moderate dysfunction. Greater variability in the low-flow group is visually obvious. These results suggest that less impaired salivary glands secrete more and also better saliva in terms of buffer capacity.

### 2.4. Minor Salivary Gland Biopsy

**Figure 5:**
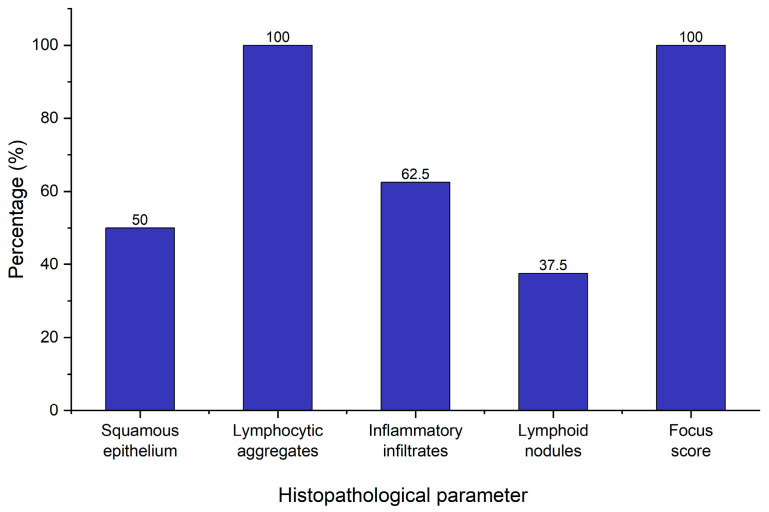
Histopathological evaluation of minor salivary gland biopsies.

As a part of the diagnostic workup for pSS, eight patients with an absence of Anti-Ro/Anti-La who fulfilled the symptomatologic criteria underwent minor salivary gland biopsy in order to confirm their diagnosis. This took place in the dental office under local anesthesia with no adverse reactions or complications during or subsequent to the procedure. All biopsies yielded adequate samples for pathologic analysis. The key histopathological parameters evaluated included the presence of squamous epithelium, lymphocytic aggregates, inflammatory infiltrates, lymphoid nodules, and the focus score—a crucial component in confirming the diagnosis (Figure 5).

All eight patients demonstrated lymphocytic aggregates (100%), and all samples showed a focus score greater than 1 (more than 1 lymphocytic cluster per 4 mm^2^ of glandular tissue), meeting the histopathological threshold stipulated by the 2016 ACR/EULAR classification criteria for pSS. These results suggest an autoimmune sialadenitis condition consistent with Sjögren’s pathology. On the other hand, a number of parameters varied across the samples. In four out of eight patients (50%), squamous epithelium was present. This is often associated with mucosal contamination during sampling and does not preclude diagnosis or other data interpretation. Inflammatory infiltrates were present in only five patients (62.5%) suggesting that while lymphocytic foci are consistently observed (focus score > 1), inflammatory background changes are less prevalent. Interestingly, lymphoid nodules were observed in four patients (50%), indicating the histopathological diversity of salivary gland inflammation in pSS. A focus score greater than 1 often coexists with variable inflammatory infiltrates and nodular structures, supporting the concept that the focus score alone may not fully depict the complexity of salivary gland inflammation.

### 2.5. Correlations Between Serological Biomarkers

**Figure 6:**
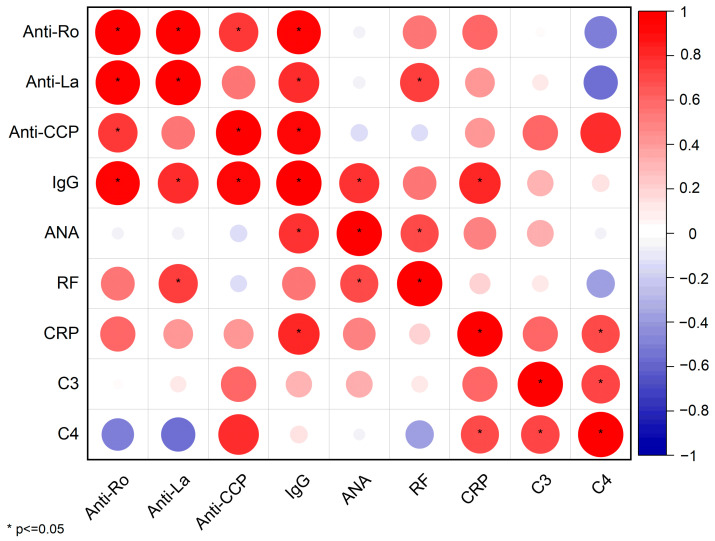
Correlation matrix between therapeutic approaches and serological biomarkers.

In order to identify statistically significant relationships among serological biomarkers, we constructed a Pearson correlation matrix (Figure 6).

This matrix outlines both the strength and direction of associations through color and circle size and also flags statistically significant correlations (*p* ≤ 0.05) with an asterisk. 

Several key immunopathological insights emerge, as follows.

*Anti-Ro/SSA and Anti-La/SSB*: These classical autoantibodies exhibit an almost perfect positive correlation (r ≈ 1; Figures S1 and S2), confirming their frequent co-occurrence in patients with pSS.

*Anti-La/SSB, IgG, and RF*: Anti-La/SSB titers also demonstrate strong correlations with total IgG (r = 0.81, *p* = 0.02) and RF (r = 0.75, *p* = 0.03), suggesting that hypergammaglobulinemia and systemic inflammation share overlapping B-cell–driven pathways.

*IgG* displays a robust positive correlation with several biomarkers, including Anti-CCP (r = 0.92, *p* = 0.008), ANA (r = 0.79, *p* = 0.03), and CRP (r = 0.84, *p* = 0.01), underscoring its central role in the pSS pathogenesis.

*C4 complement* has notable negative correlations with both Anti-Ro/SSA (r = −0.52, *p* = 0.24) and Anti-La/SSB (r = −0.58, *p* = 0.18) autoantibodies, indicating that higher titers of this biomarkers are associated with lower levels of complement C4. The complement cascade is activated by the high levels of autoantibodies, leading to the consumption of complement proteins.

On the other hand, ANA exhibits relatively weak correlations with other biomarkers, with the exception of a positive correlation with IgG (r = 0.79, *p* = 0.03). This suggests that ANA positivity is very frequent in pSS and thus has limited statistical significance across patient groups or sub-groups. Additionally, it does not give specific insight regarding disease activity of systemic inflammation.

### 2.6. Correlations Between Serological Biomarkers and Treatment

**Figure 7:**
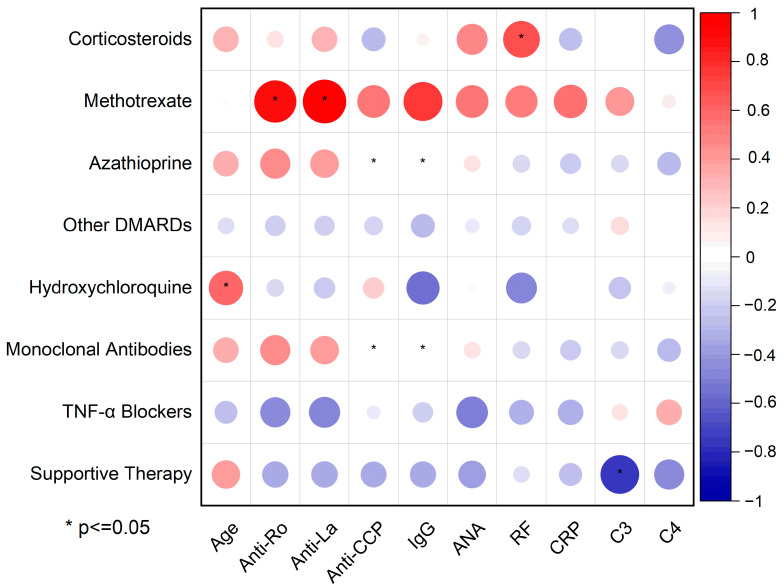
Correlation plot between therapeutic approaches and serological biomarkers.

Pearson’s correlation matrix (Figure 7) maps the linear relationships between treatment modalities—from supportive care to immunosuppressive agents—and key serological biomarkers in pSS, using circle size and a red–blue color gradient to indicate the magnitude and directionality of association (positive in red, negative in blue).

A conventional disease-modifying antirheumatic drug (DMARD), *methotrexate*, showed the strongest (circle radius > 0.7) (Figures S3 and S4) and statistically significant (* present) correlations with two serological biomarkers: Anti-Ro/SSA and Anti-La/SSB. These are the two cornerstone biomarkers involved in the immunological diagnosis of pSS, suggesting its preferential use in seropositive sub-phenotypes.

*Hydroxychloroquine* is another key treatment prescribed to alleviate fatigue, arthralgia, myalgia, and mild systemic symptoms in patients with pSS. Its use correlated positively with age (r = 0.61, *p* ≤ 0.05), indicating a preferential use in older patients. This is because hydroxychloroquine is generally considered safe and well-tolerated by patients, which makes is suitable to use in older individuals. The negative or absent correlations with Anti-Ro/SSA (r = −0.16, *p* = 0.7), Anti-La/SSB (r = −0.24, *p* = 0.57), and CRP (r = 0.01, *p* = 0.96)—even though not statistically significant—could reflect hydroxychloroquine use in less inflammatory or seronegative phenotypes. 

*Azathioprine and monoclonal antibodies* are not significantly correlated with Anti-CCP (r = 0, *p* ≤ 0.05) and IgG levels (r = 0, *p* ≤ 0.05). This suggests that these two biomarkers do not play a role in determining whether a patient is prescribed azathioprine or monoclonal antibodies. Interestingly, TNF-α blocker usage shows negative correlations with both Anti-Ro/SSA (r = −0.47, *p* = 0.24) and Anti-La/SSB antibodies (r = −0.49, *p* = 0.22). Anti-TNF-α medication is mainly used in seronegative patients and is not first-line for pSS. Examples of monoclonal antibodies used for pSS treatment include rituximab, belimumab, and abatacept. Even though TNF-α blockers (except etanercept) are monoclonal antibodies, they were analyzed separately due to their different pattern of correlation.

The most striking and statistically significant inverse correlation is found between supportive therapy and C4 depletion (r < −0.8, *p* ≤ 0.05). Supportive therapy includes artificial tears for xeropthalmia, chewing gum for xerostomia, and non-steroidal Anti-inflammatory drugs for joint pain. Without immunosuppressive agents (pathogenetic treatment), only symptomatic therapy failed to control the disease, resulting in decreased C4 levels. In other words, a low C4 level—potentially indicative of active disease—appears to be the result of insufficient use of immunosuppressive medication, which may have led to suboptimal disease control.

## 3. Discussion

This study was undertaken to fill critical gaps in our understanding of primary Sjögren’s syndrome within an Eastern European setting. Specifically, we aimed to characterize the demographic, functional salivary, serological, and histopathological features of Romanian pSS patients and map the interrelationships among key biomarkers, glandular function, and treatment choices. By combining noninvasive salivary assays, minor gland biopsy data, and a comprehensive serological panel, we sought to develop an integrated, biomarker-driven framework to guide diagnosis and personalized management in a real-world cohort.

Our cohort—primarily middle-aged to older adults (mean age 52.8 years)—reflects the classic epidemiology of pSS, with a strong female predominance (F/M = 7:1) and an age profile consistent with prior reports (53 years [10]; 50 years [11]; 54 years [12]).

Since the ACR/EULAR 2016 diagnostic criteria require a score of at least 4 points from weighted items, salivary flow studies alone do not achieve the diagnostic threshold for pSS in seronegative patients. The salivary flow studies in this cohort highlight a high prevalence of glandular dysfunction among patients with pSS. Over 60% of patients had drastically reduced unstimulated salivary flow (rates below 0.1 mL/min), and a notable proportion also presented acidic salivary pH and decreased buffering capacity. This percentage is highly influenced by the criteria used to select the patients involved in the study. For example, other authors reported proportions of patients with UWSFRs less than 0.1 mL/min of 52% [13] and 83% [14]. However, this ratio can vary from cohort to cohort and is not considered a standard. The salivary pH is reduced in pSS patients, indicating a diminished protective effect against dental caries and tooth decay. This observation is in agreement with data reported by other authors [15,16]. Salivary tests point out their clinical utility not only in confirming glandular hypofunction but also in characterizing the severity of salivary gland impairment in pSS. Hence, a comprehensive and noninvasive assessment of exocrine impairment and oral health risk should combine flow rate, pH, and buffer capacity measurements.

Minor salivary gland biopsy remains indispensable in seronegative patients with high clinical suspicion of pSS, as it enhances diagnostic certainty and facilitates appropriate management. Every specimen in our series demonstrated a focus score > 1, thus satisfying the 2016 ACR/EULAR threshold and mirroring similar observations (all focus scores > 1) reported elsewhere [17]. Integrating these histopathological findings with functional salivary metrics enhances diagnostic certainty and captures the full spectrum of immune-mediated glandular damage.

As in most autoimmune diseases, pSS is associated with circulating autoantibodies directed against self-proteins [18]. The serological biomarker Pearson’s correlation matrix revealed significant association patterns among autoantibodies, IgG, CRP, and complement components. The strong correlations between Anti-Ro/SSA, Anti-La/SSB, IgG, and CRP highlight the interconnection between B-cell hyperactivity and systemic immune activation, as previously reported [19]. The inverse relationship between complement components and the main pSS autoantibodies supports their value in assessing disease progression and severity. All patients received treatments aligned with the Romanian standard of care, consistent with EULAR and other international guidelines. However, newer agents—such as biologics and JAK inhibitors—remain largely inaccessible due to the absence of national reimbursement; in select patients, we nonetheless administered these therapies under special approval (only five patients; 15.6%). Further, we cross-analyzed the serological biomarkers and treatment options to better understand the reasoning behind medical decisions; statistical correlations between serological biomarkers and treatment point out the quantitative relationships between pharmacologic interventions and patients’ immunologic and inflammatory profiles. Despite the undoubted utility of the correlation matrix, therapeutic decisions are influenced by multiple, often puzzling variables such as disease duration, comorbidities, and physician preference, which are too complex to be comprehended in this analysis. Future studies should consider multivariate statistical modeling approaches to adjust for potential confounders. Nevertheless, this approach is a compelling visual and statistical summary of the relationship between serological markers and treatment strategies in pSS. The most obvious patterns are methotrexate use in seropositive patients and supportive therapy in mild cases, but these also raise questions about uncharted associations.

No significant sex differences emerged in biomarker levels or treatment regimens; the ANOVA of ANA titers (α = 0.05) confirmed equivalent means. The older age of male patients may indicate later onset or diagnostic delay, meriting targeted investigation into sex-specific disease trajectories. 

Correlating our serology–treatment framework with anatomical classifications—such as Aničin et al.’s sialendoscopy-based Wharton’s duct papillae grading [20]—could elucidate how duct morphology influences salivary flow and symptom severity. Going forward, larger prospective multicenter studies incorporating molecular profiling, standardized salivary assays, and advanced statistical modeling will be essential to validate these findings and drive precision medicine approaches in pSS.

This study makes a valuable input to clinical rheumatology and oral immunopathology by demonstrating how functional salivary assays—when integrated with serological biomarkers—can inform immunophenotype-driven treatment stratification (e.g., methotrexate, hydroxychloroquine, TNF inhibitors). Ophthalmological evaluation, including patient-reported dry eye symptoms and Schirmer’s I testing, is a cornerstone of the diagnostic work-up and was performed in all participants, but these data were not included in our quantitative analyses. By embracing a precision medicine framework, our approach lays the groundwork for predictive risk models and more personalized pSS management.

Despite its contributions, this study has several limitations. The single-center, retrospective design and modest sample size (n = 32 pSS cases) constrain both the statistical power and generalizability; our correlations should therefore be interpreted with caution, as patient characteristics and methodologies may vary across settings. We also did not perform molecular-weight–based discrimination of Anti-SSA antibodies (i.e., Anti-Ro60 vs. Anti-Ro52), relying instead on standard serological assays that do not differentiate these isoforms. Moreover, although ANA patterns and titers carry significant diagnostic and prognostic value, these data were not collected. Addressing these gaps—by incorporating detailed molecular profiling of autoantibody subtypes and comprehensive ANA characterization—could illuminate disease heterogeneity and refine risk stratification. Larger, prospective multicenter studies with these enhanced serological and molecular assessments are therefore urgently needed to validate and extend our findings across diverse primary Sjögren’s syndrome cohorts.

## 4. Materials and Methods

### 4.1. Patient Cohort

This retrospective cross-sectional study involved 32 consecutive patients diagnosed with pSS (fulfilling the ACR-EULAR 2016 classification criteria for Sjogren’s syndrome). This study was conducted in Iași, northeast Romania. The patients were diagnosed and periodically examined in Clinical Rehabilitation Hospital, Iași, 2nd Rheumatology Clinic. Salivary tests and minor salivary gland biopsies were conducted in St. Spiridon Hospital, Iași. The study period spans from January 2022 to January 2024, and during this timeframe, all patients had at least one monitoring visit at the 2nd Rheumatology Clinic of Clinical Rehabilitation Hospital, Iași.

### 4.2. Diagnosis

The patients were diagnosed based on their clinical symptomatology (sicca syndrome with oral and ocular manifestations), salivary gland biopsy, immunologic profile, and salivary function assay (both stimulated and unstimulated whole salivary flow, pH, buffer capacity). Patients diagnosed with secondary Sjogren’s syndrome associated with other autoimmune rheumatic diseases (e.g., RA, systemic lupus erythematosus) were excluded from this study. Organic symptoms, including pulmonary, renal, dermatological involvement, and lymphadenopathy, were delineated in accordance with the EULAR Sjögren’s Syndrome Disease Activity Index (ESSDAI) domains [21].

### 4.3. Data Collection

The data were collected from patients’ files following a standard protocol and include demographic data (name, birth date, age, sex), clinical data (medical history, allergies, medications, family history of diseases), biological data (related to systemic inflammation: erythrocyte sedimentation rate, C reactive protein), and immunologic data (ANA antibodies, RF, Anti-SSA/SSB antibodies, C3, C4, immunograme).

The quantities of C-reactive protein, RF, IgM, and total immunoglobulin G were quantified by the immunoturbidimetric method with the XL1000 clinical chemistry analyzer from Erba-Lacherma, Brno, Czech Republic. Antinuclear antibodies (Anti-Ro/SS-A, Anti-La/SS-B, IgG), complement C3 and C4, and RF were determined using the ORGENTEC Alegria Automated Laboratory Diagnostics, which employs the Enzyme-Linked Immunosorbent Assay (ELISA) method. Positivity thresholds for the serological biomarkers were set by the devices’ manufacturers: Anti-Ro/SS-A > 22 U/mL, Anti-La/SS-B >25 U/mL, Anti-CCP >20 U/mL, ANA > 1.2 U/mL, CRP > 1 mg/dL, RF >20 UI/mL, C3 < 88 mg/dL, and C4 < 16 mg/dL.

### 4.4. Salivary Function Assay

The stimulated and unstimulated salivary flow rates were determined using the spitting method. In order to determine the unstimulated whole salivary flow rate (UWSFR), the patient sits and is instructed to swallow existing saliva to clear their mouth. After that, the patient spits all the saliva into a pre-weighed collection tube. The volume of saliva is measured after a 5-minute collection time. For the stimulated whole salivary flow rate (SWSFR), the patient is instructed to chew a piece of wax. After 30 seconds, the patient spits into the spittoon. For the next 5 minutes, the patient is instructed to keep chewing while spiting all of the produced saliva into a pre-weighed container, at certain time intervals. The saliva pH and salivary buffering capacity were assessed with the GC Saliva-Check Buffer (Leuven, Belgium).

### 4.5. Minor Salivary Gland Biopsy Assay

The area of the lower lip selected for salivary gland tissue sampling is anesthetized with a local anesthetic (spray benzocaine). A small incision (~1 cm) is made on the lip mucosa a few millimeters away from the midline. The surgeon recognizes the minor salivary glands by their lobular structure. Several lobules (3–7 lobules) are carefully removed using a forceps and scalped blade. The incision is closed with interrupted sutures, typically absorbable sutures. The removed lobules are placed in a formalin-based container and sent to the physiopathology lab for examination. The samples are microscopically examined to assess the presence of squamous epithelium, lymphocytic aggregates, inflammatory infiltrates, and lymphoid nodules. Additionally, the focus score is determined as the number of lymphocytic foci (groups of 50 or more lymphocytes) per 4 mm^2^ of salivary gland tissue.

### 4.6. Statistics

Statistical analysis was conducted using SPSS software (version 24.0) to perform analysis of variance (ANOVA) and two-sample t-tests. The chi-square test was conducted for discrete parameters where the expected *p* values were deemed statistically significant at *p* ≤ 0.05. The resultant data were converted into graphical representations via Origin 2021. The serological biomarker data and graphics were plotted by categorizing the patients into two primary groups: below and above 50 years old. The correlation matrix among serological biomarkers and between therapeutic approaches and serological biomarkers was generated in Origin Pro 2024 using Pearson correlation coefficients with pairwise exclusion for missing data. Statistically significant correlations (*p* ≤ 0.05) are highlighted through starred entries.

### 4.7. Approval

This study was conducted in accordance with the Declaration of Helsinki and approved by the Ethics Committee of the University of Medicine and Pharmacy “Grigore T. Popa” Iasi, No. 426/7 April 2024. All patients provided written informed consent before participating in the study.

## 5. Conclusions

These results outline the dynamics between serological biomarkers, salivary tests, minor salivary gland biopsies, and treatment approaches. This study focuses especially on clinical aspects, and comprehensive statistical methods are used to point out the interrelationships between different parameters. The analysis of serological biomarkers in patients with Sjögren’s syndrome reveals significant age-related trends in immune system activity. While classical markers such as Anti-Ro/SS-A and Anti-La/SS-B maintain high positivity across both age groups, notable differences were observed in the complement components C3 and C4. On the other hand, salivary tests may help identify patients at increased risk of oral complications such as caries, candidiasis, and xerostomia-related discomfort. Salivary parameters may also serve as useful monitoring tools for disease progression or therapeutic response. There are distinct associations and treatment patterns in patients with pSS, and the correlation plot between the therapeutic strategies and serological biomarkers shines a light on this often-overlooked topic. These findings underscore the heterogeneity of pSS and support a stratified, biomarker-informed approach to treatment selection. Ultimately, such analyses can contribute to more personalized and immunophenotype-driven therapeutic approaches in the management of pSS.

## Figures and Tables

**Table 1:** Descriptive statistics of patient’s age.

**Parameter**	**Value (Years)**	**Parameter**	**Value (Years)**
Mean	52.8	Maximum	72
Standard deviation	9.9	Minimum	27
		Median	54.5
**Women**		**Men**	
Mean	51.5	Mean	61.5
Standard deviation	9.8	Standard deviation	2.8

## Data Availability

The original contributions presented in the study are included in the article, and further inquiries can be directed to the corresponding author.

## Supplementary Materials

The following supporting information can be downloaded at: https://www.mdpi.com/article/10.3390/ijms26136327/s1.
