# Supplementary material for: Comprehensive Clinical, Serological, and Molecular Biomarker Profiling of Primary Sjögren’s Syndrome: A Single-Center Cohort Study in Northeastern Romania
Source: Int J Mol Sci. 2025 Jun 30;26(13):6327. doi: 10.3390/ijms26136327 (PMC12250070; doi:10.3390/ijms26136327)
Supplement: Supplementary file 1 [file ijms-26-06327-s001.zip › ijms-3705250-supplementary.pdf]

Supporting information

Figure S1. Raw Pearson *r* values for the statistical correlation between serological biomarkers.

	Anti-Ro	Anti-La	Anti-CCP	IgG	ANA	RF	CRP	C3	C4
Anti-Ro	1.0	0.96	0.76	0.95	-0.074	0.56	0.60	0.048	-0.52
Anti-La	0.96	1.0	0.55	0.81	-0.079	0.75	0.43	0.13	-0.58
Anti-CCP	0.76	0.55	1.0	0.92	-0.15	-0.14	0.43	0.61	0.81
IgG	0.95	0.81	0.92	1.0	0.79	0.55	0.84	0.34	0.16
ANA	-0.074	-0.079	-0.15	0.79	1.0	0.71	0.52	0.35	-0.069
RF	0.56	0.75	-0.14	0.55	0.71	1.0	0.22	0.14	-0.39
CRP	0.60	0.43	0.43	0.84	0.52	0.22	1.0	0.60	0.71
C3	0.048	0.13	0.61	0.34	0.35	0.14	0.60	1.0	0.72
C4	-0.52	-0.58	0.81	0.16	-0.069	-0.39	0.71	0.72	1.0

Figure S2. *p* values for the statistical correlation between serological biomarkers.

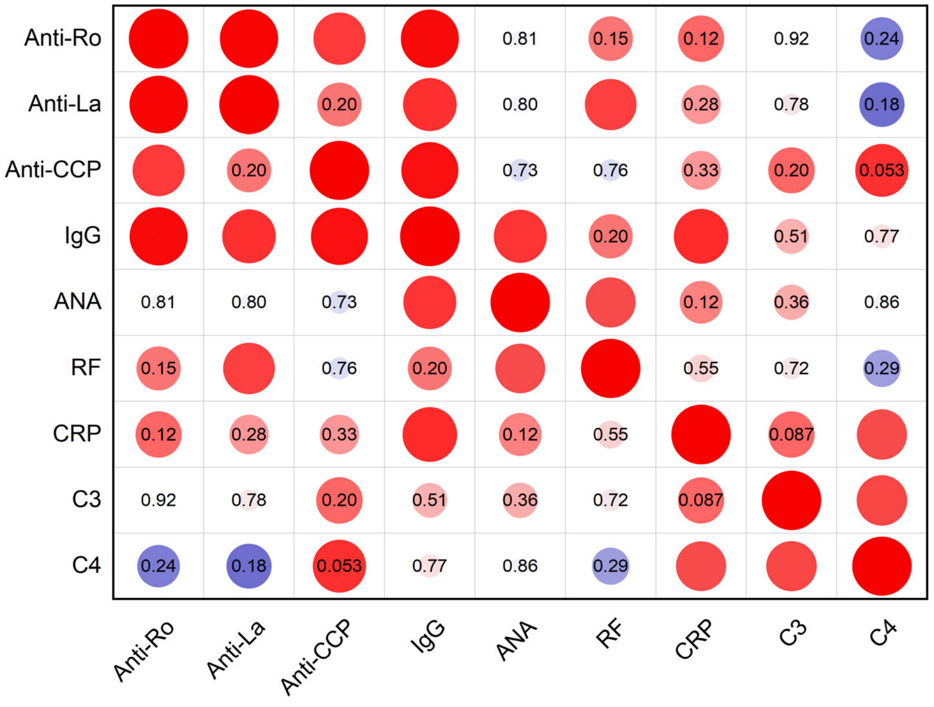

Siginficant level: 0.05

Figure S3. Raw Pearson *r* values for the statistical correlation between serological biomarkers and treatment.

	Age	Anti-Ro	Anti-La	Anti-CCP	IgG	ANA	RF	CRP	C3	C4
Corticosteroids	0.34	0.15	0.33	-0.29	0.083	0.49	0.69	-0.27	-0.019	-0.46
Metotrexat	-0.026	0.91	0.97	0.55	0.78	0.55	0.53	0.57	0.43	0.10
Azatioprin	0.34	0.47	0.42			0.14	-0.17	-0.22	-0.17	-0.29
Other DMRADs	-0.14	-0.22	-0.21	-0.19	-0.29	-0.11	-0.20	-0.14	0.17	-0.0024
Hydroxychloroquine	0.61	-0.16	-0.24	0.24	-0.56	0.048	-0.49	0.019	-0.26	-0.083
Monoclonal Antibodies	0.34	0.47	0.42			0.14	-0.17	-0.22	-0.17	-0.29
TNF-alfa blockers	-0.27	-0.47	-0.49	-0.10	-0.22	-0.52	-0.32	-0.33	0.14	0.34
Supportive Therapy	0.42	-0.36	-0.36	-0.35	-0.35	-0.39	-0.15	-0.27	-0.77	-0.47

Figure S4. *p* values for the statistical correlation between serological biomarkers and treatment.

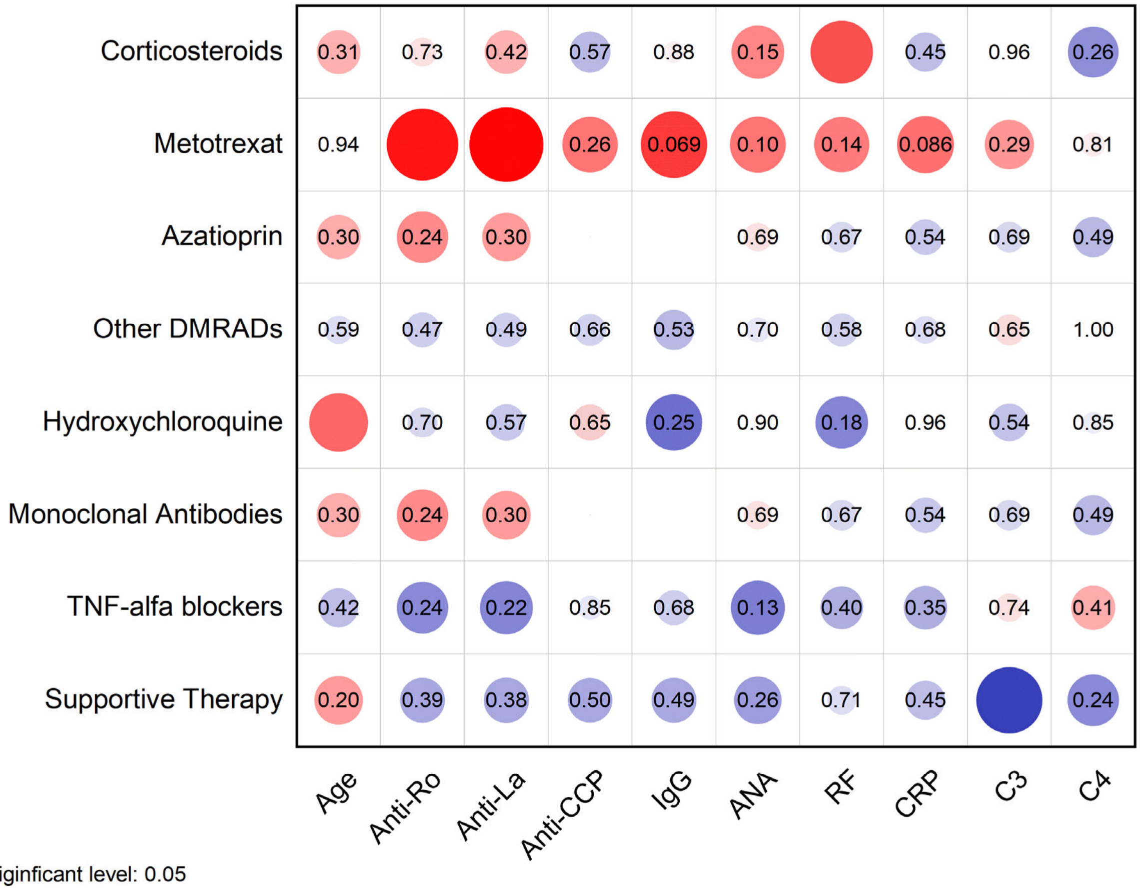

Sigintficant level: 0.05
